# Impact of genetic variation and geographic distribution of porcine reproductive and respiratory syndrome virus on infectivity and pig growth

**DOI:** 10.1186/1746-6148-9-58

**Published:** 2013-03-27

**Authors:** Bouabid Badaoui, Roberto Grande, Stefano Calza, Maria Cecere, Mario Luini, Alessandra Stella, Sara Botti

**Affiliations:** 1 Parco Tecnologico Padano - CERSA, Via Einstein, Lodi, 26900, Italy; 2 Università degli Studi di Brescia, piazza del Mercato, Brescia, 25121, Italy; 3 Istituto Zooprofilattico Sperimentale della Lombardia e dell’Emilia Romagna, Lodi, 26900, Italy

**Keywords:** PRRSV, ORF5, ORF7 sequences, Glycosylation

## Abstract

**Background:**

The porcine reproductive and respiratory syndrome (PRRS) is a devastating disease for the pig industry. In this study, we analysed the genetic variability of PRRS virus (PRRSV) as well as the relationship between the genetic variability, the geographical and temporal distribution of the PRRSV strains. Moreover, we investigated the association between the glycosylation patterns in PRRSV sequences and pigs growth.

**Results:**

The data highlight that PRRSV strains evolve rapidly on individual farms, and temporal evolution of PRRSV is an important factor of genetic variability. Analysis of glycosylation sites in the glycoprotein 5 (GP5) ectodomain revealed that PRRSV isolates had seven combinations of putative N-linked glycosylation sites of which the N37/46/53 sites was found in 79% of the sequences. No significant relationship was found between the genetic variation of the PRRSV strains and the geographic distance. A significant relationship was found between the genetic variation and time of sampling when farm was considered as a factor in the analysis. Furthermore, the commercial semen from artificial insemination centres was not a source of PRRS transmission.

The PRRSV having the glycosylation site at position N46 (N46+) were observed to have higher burden on pigs and accordingly the corresponding infected pigs had lower average daily gain (ADG) compared with those infected with PRRSV lacking the glycosylation at N46 (N46-) position site. This study showed that the number of piglets by litter infected by PRRSV was lower for the Landrace breed than for the other studied breeds (Large White, Duroc and Pietrain).

**Conclusions:**

The PRRSV genetic variability which is determined by a local and temporal evolution at the farm level could be considered in a perspective of prevention. Moreover, the association between the PRRSV glycosylation patterns and its virulence could be of interest for vaccine development. The differences of resistance to PRRSV infections among pig breeds might open new horizons for the genetic selection of robustness against PRRSV infection.

## Background

Porcine reproductive and respiratory syndrome (PRRS) is a devastating swine disease [1] associated with reproductive failure, growth decrease [2] and a drop in spermatogenesis [3]. PRRSV is a member of Arteriviridae virus family, in the order of Nidovirales, and it has a 15 kb single stranded RNA genome that encodes nine open reading frames (ORF). The ORFs 1a and 1b encode the non-structural proteins Nsp1a, Nsp1b, and Nsp2-12, while ORF2a, ORF2b, ORFs 3–7 and ORF5a encode the structural proteins GP2a, GP2b, GP3, GP4, GP5, M, N and ORF5a-protein, respectively [4-6].

PRRS viruses have high biological diversity, and two main genotypes are recognised: the genotype 2 (North American) and genotype 1 (European) types [7]. All Italian PRRSV strains were of genotype 1 and exhibited high nucleotide variability [8-10]. PRRSV can be transmitted via semen [11], contact between pigs [12], contaminated transport vehicles [13] and aerial spread [14]. Clarification of the mechanisms of the virus spread and differentiation by study of the relationship between genetic, geographical and temporal variability of PRRSV may help in the development of better strategies to control the PRRSV spread. [15].

In order to study the genetic basis and the epidemiology of PRRSV we monitored 18 farms in Northern Italy through the period 2006–2009 (Misagen project: http://www.itb.cnr.it/misagen). We created a database collecting genotypes, phenotypes and genealogic data from pure breed pigs and established a bio-repository containing more than 20.000 pig samples. A total of 541 litters were monitored during the project.

In this study we compared the nucleotide variability among PRRSV strains isolated during the three years project by sequencing samples from all 266 litters that where positive for PRRSV. Therefore, we analysed the relationship between the PRRSV genetic diversity from one side and the geographical and temporal distances of the PRRSV sampling from the other side. Moreover the effect of the glycosylation pattern of the PRRSV GP5 on the pathogenicity of the virus and on pigs ADG within different breeds was studied.

## Methods

### Sampling

**Figure 1:**
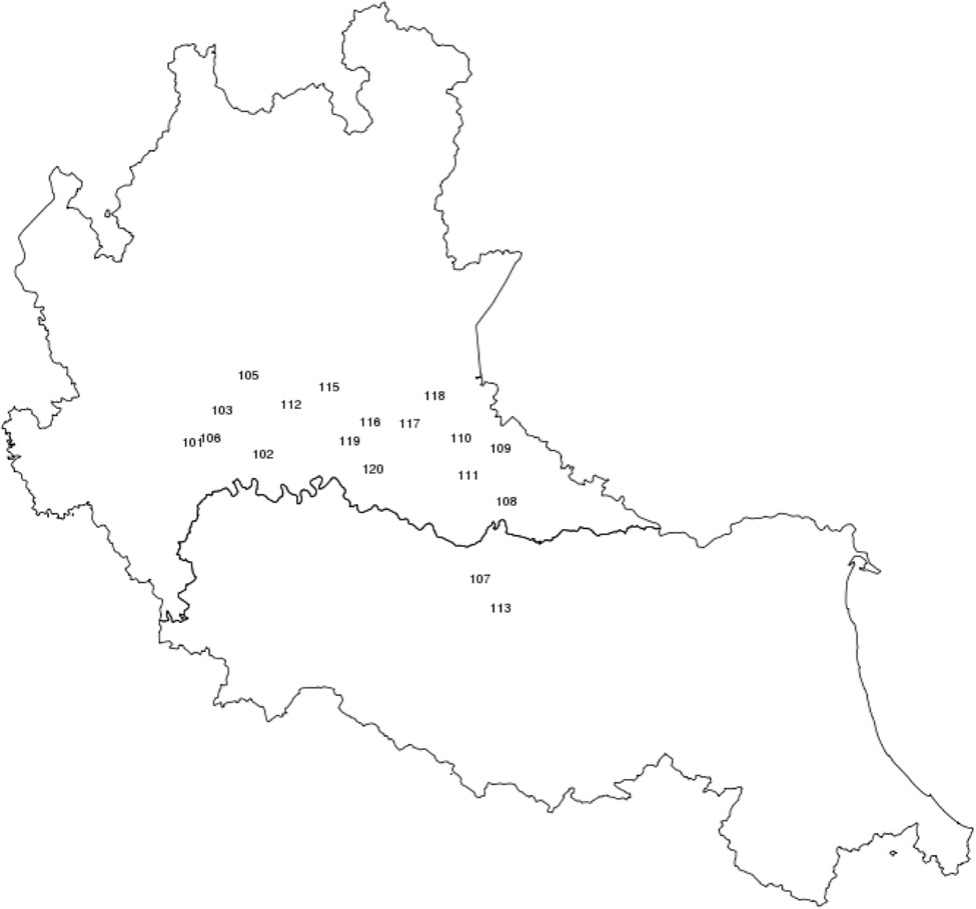
**Map showing the position of the farms from which serum samples were collected.** The numbers correspond to 18 farms located in the north of Italy: 16 farms are in the Lombardy region and 2 farms are in Emilia Romagna.

Serum samples were collected by veterinarians from different farms located in the North of Italy (*Brescia*, *Lodi*, *Cremona*, *Mantova* and *Reggio Emilia)*. Representative farms rearing purebred pigs (Large White, Landrace, Duroc, and Pietrain) in Lombardy region were chosen based on size, housing, and management (Figure 1).Farms were included in the project if they were positive for PRRSV and they had at least two pure pig breeds. In each farm, veterinarians took serum samples from at least 4 piglets from each litter. Serum samples collected during the weaning phase (45–50 day post-partum) were used for the analyses. For each animal, the ADG (in kg/day) was calculated from 5–10 to 25–30 days after birth, defined as ADG1 and from 25–30 to 45–50 after birth and defined as ADG2. All samples were collected between January 2006 and March 2009. Samples were added to the PRRS bio-repository [16] which presently contains more than 20,000 samples collected from pigs in these farms.

### RNA extraction, PCR diagnostic and nucleotide sequencing

Viral RNA was extracted immediately from serum using High Pure Viral RNA Kit (Roche Diagnostics GmbH; Mannheim, DE) and stored at −80 °C. Presence or absence of PRRSV was determined by multiplex PCR of conserved regions of viral ORF7 using primers and conditions previously described [10,17]. One positive sample per litter was chosen to analyse the RNA sequence. PCR conditions for sequence all ORF5 and ORF7 were previously described [18,19]. PCR products were sequenced using the Big Dye Terminator Cycle Sequencing kit (Applied Biosystems; Foster City, US) following the manufactories instructions, run and analysed on an ABI 3730 DNA Analyser (Applied Biosystems; Foster City, US). All products were sequenced in both directions. This study was exempt as serum sampling from pigs is a part of routine care and does not need an ethical approval.

### Analysis of sequences, phylogenetic relationships and N-glycosylation sites

Sequences were compared with the Lelystad virus (LV, Genebank accession number *M96262*) which was used as a reference using Bioedit (http://www.mbio.ncsu.edu/BioEdit/bioedit.html)*,* sequence alignments were carried out using ClustalX [20]. We used the MODELGENERATOR [21] program to construct the phylogenetic trees for ORF5 and ORF7. This software spans through 56 different models for phylogenetic trees constructions. In this analysis, the best model was chosen based on likelihood ratio. The best fitting tree was estimated by feeding into the program PHYML [22] the input parameters from MODELGENERATOR. Genetic distances between aligned sequences were estimated according to a specific substitution model using Tree-Puzzle [23].

The entropy approach [24], which measures the uncertainty at each position relative to other positions in a specific alignment, was used to assess the variability along the ORF5 and ORF7 sequences. The N-glycosylation sites in ORF5 sequences were identify using the NetNGlyc 1.0 software [25].

### Spatial and temporal trend analysis

Latitude and longitude coordinates were used to calculate the distance between farms in kilometres (km) using the great-circle formula [26]. In every analysis one sequence from each litter was selected by random sampling.

Correlation between genetic and spatial distances was calculated using the Mantel correlation statistic (r_M_) based on the Spearman correlation coefficient [27]. As values in the distance matrix were not independent, the test was based on a permutation procedure using 1000 random re-samplings. The partial Mantel test was used to compare two distance matrices correcting for the third one [27]. P-values for the Mantel tests are one-sided, i.e. the null hypothesis assumes r_M_ equal to 0.

In order to account for the presence of several piglets from the same farm and therefore to remove the farm clustering effect, a bootstrap estimate with 1000 re-sampling of r_M_ was calculated using sequences from each farm. The average correlation coefficient for data stratified within farms was calculated following [28].

### Analysis of PRRSV transmission by semen

The effect of commercial semen on PRRS virus transmission was evaluated using a permutation test MANOVA [29,30] accounting for the farm and semen source as effects. P-values were calculated based on 1000 permutations.

All the statistical analysis were performed using the R environment (R Development Core Team) [31] using functions implemented in the packages vegan, ecodist and ape.

### Associations between PRRSV glycosylation patterns, PRRSV infectivity and pigs ADG

The percentage of PRRS positive piglets in each litter, determined by diagnostic PCR was used as an estimate of the PRRSV infection capacity. Two PRRSV glycosylation patterns of the ORF5 were considered: 1) ORF5 with N-linked glycosylation site N46 (N46+) and 2) ORF5 without N-linked glycosylation site N46 (N46-). Interestingly, the N46 glycosylation site has been found to be associated with the PRRSV infectivity [32,33].

Association analyses were performed using the general linear model Y = Xβ + e, implemented in the R statistical software. Y represented either a quantitative variable (percentage of infected piglets, or ADG) or a binomial variable (pattern of glycosylation). β is the vector of fixed factors which included: farm, breed effects, and depending on the analysis, also included either the percentage of infection and/or ADG. X is the matrix of indices for the fixed effects; e is a vector of the residual random errors, which were assumed to be normally distributed. Correlations among quantitative variables (infection percentages and ADG) were estimated using the Pearson method.

## Results

### Phylogenetic analysis and clustering of sequences ORF5 and ORF7

Using the diagnostic PCR a total of 266 litters was positive for the PRRS virus. A total of 248 ORF5 sequences from 235 litters and 288 ORF7 sequences from 266 litters were generated. The ORF5/ORF7 sequences pertain to Large White (56 %), Duroc (21-22 %), Landrace(17-18 %) and Pietrain (5 %) breeds. The ORF5 and ORF7 sequences came from 17 and 18 farms, respectively. The average distance between farms was 54.0 km, ranging from 6.06 km to 122.25 km.

**Figure 2:**
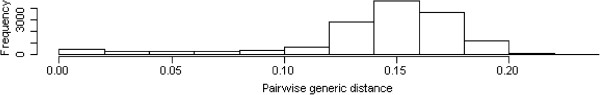
**Neighbour-joining tree of ORF5 of Italian PRRSV strains generated in this study (red colour) and three European PRRSV vaccines retrieved from NCBI databases.** The green, yellow and blue colours correspond to the vaccines DQ324668, DQ324681 and DQ324678, respectively. The circle highlights the Italian strains that clustered with the vaccines.

The sequences for ORF5 and ORF7 were 606 and 387 nucleotides, respectively. To construct the corresponding phylogenetic trees, two different nucleotide substitution models were used for ORF5 and ORF7. For ORF5 the nucleotide substitution model HKY [34] gave the best fit, while for ORF7 the best model was the K2P + I + G (Kimura’s two-parameters model, K2P)[35]. A distance matrix calculated using HKY MODELGENERATOR estimated a transition/transversion ratio of 5.80, a proportion of invariable sites as 36% and nucleotide frequencies: A = 23.17 %, C = 25.44 %, G = 23.64% T = 27.75 %. A discrete-gamma model with 4 categories was used to account for the variable substitution rates among sites, with shape parameter 0.89. Similarly for the K2P model, a transition/transversion ratio of 9.17, a proportion of invariable sites of 29% and a gamma shape of 0.44 were used. The trees resulting from maximum likelihood optimization for ORF5 and ORF7 respectively showed a strong clustering by farm (Figures 2A and 2B).

**Figure 3:**
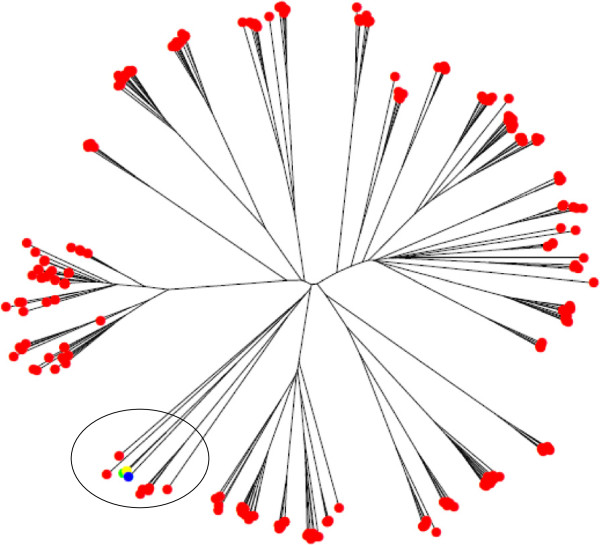
**Neighbour-joining tree of A: ORF5 (N = 248) and B: ORF7 (N = 288) of PRRSV virus isolates used in this study as well as Lelystad strain (Genbank accession number M96262).** In brackets the sample provenance is reported (farm number). One part of each tree was zoomed to show the strong clustering of ORF5 and ORF7 by farm.

All ORF7 sequences were of European genotype PRRSV-1 having coding for a protein of 128 amino acids. Phylogenetic trees for the ORF5 and ORF7 split the PRRSV isolates mainly depending on farm which might suggest the combination of PRRS strain (“founder effect”) and farm effect on PRRSV strains evolution. ORF5 and ORF7 had entropy of 0.0112 and 0.0083, respectively and showed 13 and 17 conserved regions, respectively (Additional file 1A and 1B). To evaluate the genetic diversity of the PRRSV Italian strains, we used the average pairwise genetic distance (APD) between ORF5 sequences of the PRRSV isolates, a standard methodology usually used for this scope [9,36]. The PRRSV strains showed an average pairwise distance (APD) equal to 0.15 (Figure 3). This value is higher than that reported for other European strains [37].

### Identification of ORF5 glycosylation sites

The Lelystad reference PRRS virus has two glycosylation sites on the GP5 protein: “N46” and “N53”. Approximately, 79% (196 out of 248) of the Italian isolates sequenced in this study carried three glycosylation sites at positions N37, N46 and N53. Around 4% (10 out of 248) had the first glycosylation site at position N36 and other two sites at N46 and N53. Only 0.16% (4 out of 248) of the isolates had the 2 glycosylation sites of the Lelystad strain (Table 1). Interestingly 15% (38 out of 248) were missing the glycosylation site at position 46, changing the asparagine to aspartic acid. A similar distribution of glycosylation sites was found in 97 Italian sequences present in NCBI (data not shown) in which 73% had the glycosylation sites at positions N37, N46 and N53, with only 3% missing the glycosylation site at position N46.

**Table 1:** Glycosylation pattern in different pig breeds (Duroc, Large white, Landrace and Pietrain)

**Glycosylation**										**Number of animals per breed carrying the specific glycosylation type**				
**Glycosylation Sites**									**Type**	**Duroc**	**Large White**	**Landrace**	**Pietrain**	**Total**
.	.	37	.	.	46	.	.	53	AA	46	109	34	7	196
.	.	37	.	.	.	.	.	53	BB	9	10	9	4	32
.	36	.	.	.	46	.	.	53	CC	1	8	1	0	10
.	.	.	.	.	46	.	.	53	DD	0	3	0	1	4
.	.	.	.	.	.	.	.	53	EE	1	0	2	0	3
35	.	.	44	.	.	51	.	.	FF	0	1	0	1	2
.	36	.	.	45	.	.	52	.	GG	0	1	0	0	1
									Total	57	132	46	13	248

Note the domination of the AA glycosylation pattern in all the breeds.

Seven glycosylation patterns (AA, BB, CC, DD, EE, FF and GG) have been found in Italian breeds. Note the domination of the AA glycosylation pattern in all the breeds.

### Correlations between genetic, geographical and temporal distances

The correlation between PRRSV strains genetic distance and the geographic distance between the sites from which the isolates had been sampled were significant for both ORF5 and ORF7, (ORF5: r_M_ = 0.170, p-value = 0.001, ORF7: r_M_ = 0.166, p-value = 0.001) when we did not consider the farm effect in the model. To account for the effect of the farm and collection time disparities (sampling was over a 3 year period), a partial Mantel test was used to correct for farm and time. The associations between the genetic distance and the geographical distance were not statistically significant either for ORF7 (r_M_ = 0.03, p-value = 0.13) or ORF5 (r_M_ = 0.02, p-value = 0.2). This suggests that the positive correlation between the genetic distance and geographical distance might be a function of within farm clustering. To test the latter hypothesis, a permutation test was performed that randomly sampled one sequence from each farm and calculated the corresponding r_M_ (the null hypothesis assumes r_M equal_ to 0). The median r_M_ was not significantly different from zero (p-value < 0.05) which means that there is no significant correlation between the genetic and geographical distances when the farm factor was taken into consideration.

Using the same approach, the relationship between temporal (sampling times) and genetic distances was assessed. A partial Mantel correlation was performed to account for the farm (coded as 0 if from the same farms or 1 if from a different farm). Furthermore, to allow for a sufficient number of permutations only farms with at least 7 sequences were considered. In this case ORF5 and ORF7 genetic diversity is highly correlated with sampling time (ORF5 r_M_ = 0.35, p-value < 0.001, ORF7 r_M_ = 0.49, p-value = 0.001).

### PRRSV transmission by semen

Semen as a source of PRRS was evaluated using a multivariate ANOVA where the variability in PRRS sequence on a farm were correlated with semen vendor used by that farm. If one or more semen producer is a source of PRRSV contamination we would expect that the PRRSV sequences extracted from piglets which were born from such semen would be more similar than other sequences originated from piglets born from another semen producer. Four semen producers were used by the farms sampled, though 55% of the semen was of local farm origin as the main source of sequence clustering is the farm itself, in the model we accounted for a farm effect. No significant effect was found either for ORF5 (p-value = 0.36) or ORF7 (p-value = 0.64).

### Associations between glycosylation patterns, PRRSV strains infectivity and pig growth

The glycosylation site N46 was associated with ADG1, although this was not statistically significant (p =0.09). Animals infected with PRRSV strains glycosylated at position N46 (N46+) had a lower ADG1 (0.189 kg/day) than animals infected with PRRSV strains lacking this glycosylation (N46-) site (0.212 kg/day). The Pietrain breed that was present mainly in one farm with another two breeds, was infected preferentially by PRRSV N46+ (p = 0.016, ANOVA Model corrected for farm effect). However, this finding needs to be confirmed with larger sample of the Pietrain breed.

No significant association was found between the glycosylation site N46 and the percentage of infected pigs in the litter. Using the Pearson correlation, we found that ADG2 was positively correlated to the percentage of piglets infected by PRRSV N46- strains (r = 0.36) but not in the animals infected by PRRSV N46+ strains (r = −0.06). Moreover, the percentage of Landrace pigs infected with PRRSV was low compared with the other breeds (Large White, Duroc and Pietrain) (p =0.08), suggesting a higher resistance in this breed to PRRSV infection.

## Discussion

In this study, we used the PRRSV ORF5 and ORF7 sequences to assess the effects of geographical and temporal sampling on PRRSV genetic diversity from 266 litters that where positive to PRRSV. In addition, the effect of the glycosylation pattern of PRRSV GP5 on infectivity of the virus, pigs ADG and breed resistance/susceptibility to PRRSV infection was studied.

No significant correlation between the genetic and spatial distances of the Italian PRRSV ORF5 and ORF7 sequences was found. This suggests that, in farms and during the study period, PRRSV was not easily transmitted between neighbouring farms. We did not observe any acute PRRSV outbreak in the sampling period. If such outbreaks had occurred, relationships between genetics and geography may also have been positive and significant.

Our results are in contrast with previous works [14,38,39] but in concordance with the work reported by Goldberg et al. [15] and suggest that in northern Italy, PRRSV is most frequently spread among farms by the long distance, transportation of animals or semen. To confirm this latter hypothesis, we evaluated the possible effect of commercial semen source on PRRSV transmission. At least for the artificial insemination centres considered in this study, no significant effect of commercial semen on PRRSV transmission was found for neither ORF5 nor ORF7. Nevertheless, Weigel et al. [40] reported that artificial insemination increases significantly the risk that a farm is infected by PRRSV.

We found that the genetic diversity of both ORF5 and ORF7 was highly correlated with time of sampling due to evolution of the local strains (Figure 2A and 2B). Our result is in contrast with the findings by Goldberg et al. [15] and Mondaca-Fernandez et al. [14]. This might be due to a period of observation of only one year by these authors or to the continuous introduction of new variants to the observed farms.

The characterization of genetic diversity of PRRSV could be highly relevant to diagnostic testing [41], accordingly we evaluated the genetic variability in ORF5 and ORF7. All the obtained sequences were of genotype 1 and supported earlier findings on extraordinarily high diversity of Italian PRRSV compared to other countries in Western Europe [42]. Therefore, using ORF5 sequences, we reported an APD of 0.15 for the strains considered in this study. A similar result (APD = 0.157) has been reported by [42] albeit they used only 13 sequences.

**Figure 4:**
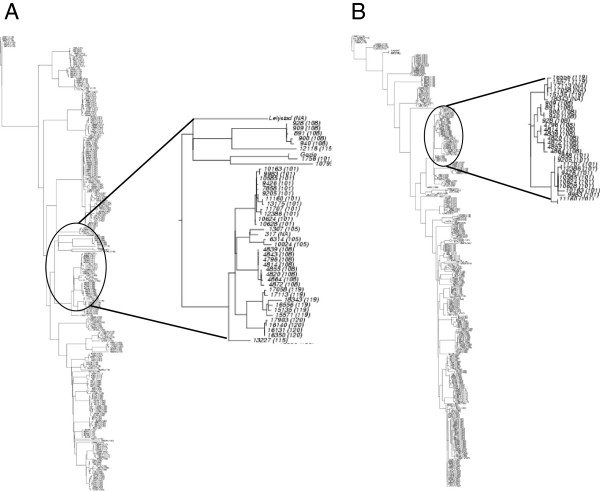
Pairwise genetic distance among PRRSV ORF5 sequences in Italian PRRSV isolates.

Modified live vaccine related strains are known to persist on vaccinated farms and to spread between farms [37]. In Italy some vaccines (e.g. Pórsilis PRRS) are registered and used but in the analysed farms these vaccines were never used to vaccine piglets. In contrast the sows were vaccinated on 50% of the farms considered in this study. However, few Italian ORF5 sequences clustered together with vaccine sequences (Figure 4), so a direct link between them and the transmission of vaccine related strains can be excluded.

The analysis of the glycosylation patterns in ORF5 in Italian strains revealed that most of them (206 out of the 248) carried three asparagines N-glycosylated at positions “N35/36/37”, “N46” and “N53”. It was suggested that the full glycosylated state is the most advantageous for virulence, easier spread and persistence in lymphoid tissue [33,43-46]. In this work we studied the effect of N46 glycosylation on the infectivity of the PRRSV strains and on the pigs ADG. The glycosylation of N46 has been shown to be fundamental to the formation of viral particles and PRRSV ability to produce infectious progeny because GP5 could undergo improper folding [32,33]. Statistical analysis showed that glycosylation pattern has an effect near significance (p-value =0.09) on ADG1 but not ADG2 (p-value 0.75). Indeed, animals infected with PRRSV N46+ had an ADG1 11% lower than that in pigs infected with PRRSV N46-. This may suggest that the N46+ glycosylation pattern impairs pig immunity and consequently pig growth in the first weeks of life.

We used the Pearson correlation to assess the correlation between the PRRSV infectivity and the pig ADG1 and ADG2. For this task, we divided the animals into two groups: 1) pigs infected with PRRSV N46+ and 2) pigs infected with PRRSV N46-. Accordingly, we found that the correlations between pigs ADG2 and the PRRSV N46+/N46- infectivity were −0.06 and 0.36 respectively. In other words, in the litters infected with N46- strain, pig growth was not inhibited while in the case of litters infected with N46+ strains, pig growth was slowed. This finding may suggest that pigs infected with PRRSV N46+ consume more energy to fight infection during 45–50 days after birth than the pigs infected with PRRSV N46-.

Associations between the breed and the glycosylation pattern would suggest a selective susceptibility/resistance of certain breeds to certain PRRSV strains rather than a casual association between glycosylation pattern and breed. Pietrain breed was infected preferentially by PRRSV N46+ (p = 0.016), however, this result is inconclusive due to the small number of pigs for Pietrain breed. Interestingly, the Landrace breed showed low PRRSV infection percentages compared with other breeds which may suggest specific resistance of this breed to PRRS infection. Ours results are in concordance with previous “*in vitro*” bstudies that evaluated the response of alveolar macrophages from five genetic lines of commercial pigs to PRRSV infection [47]. According to the latest study, Landrace breed showed a lower replication rate of the viral RNA over 30 hours and a lower viral titre at 72 h post-infection than the other breeds (Large White, Pietrain and other synthetic lines). It has been suggested that genetic factors specific to the Landrace breed could be responsible for the *in vitro* attenuation of PRRSV replication in alveolar macrophages [47].

Vincent et al. [48] reported a similar result and suggested that the slower viral accumulation and replication, resulting in reduced viral load, would enable the Landrace breed to generate a more efficient adaptive immune response. In addition, we performed in field study in which the antibody response of 1200 pigs from four breeds (Landrace, Large White, Duroc and Pietrain) to PRRSV infection was measured. Interestingly, we observed a higher antibody response to PRRSV in the Landrace and Pietrain breeds (data not published). Our results confirm previously reported findings stating that N-glycans surrounding the major neutralizing epitope located in the ectodomain of GP5 protect the virus from antibody neutralization and impair the immunogenicity of the epitope [33,43,46]. Nevertheless, PRRSV isolates lacking N-linked glycan at certain sites in GP5 grew in MARC-145 cells and infected pigs [43,49]. Similarly, [50] showed that mutation of individual glycosylation sites at N30, N35, N44 and N51 in GP5 did not affect virus infectivity in cultured cells and pig. More studies are needed to shed light on N-linked glycans effects on PRRSV pathogenicity.

To the best of our knowledge, this is the first study to analyse the association between PRRSV genetic variability (sequences and glycosylation patterns) and pig phenotypes such as PRRSV infectivity by litter and ADG.

Using sequences of PRRSV ORF5 and ORF7, we showed that the clustering of PRRSV strains was mainly determined by the farms from which the samples were collected. We found a significant positive association between the PRRS genetic variability and the sampling time distance. The absence of geographical distance and semen effect on PRRSV variability might indicate that transport via long-distance movement might be a stronger determinant of PRRSV ORF5 and ORF7 diversity. The glycosylation had an effect on ADG; in particular PRRSV N46+ seems to interfere with pig ADG. No association between the glycosylation pattern and the breed has been found except a suggestive significance (p < 0.1) for the Pietrain breed that has been found to be preferentially infected with PRRSV N46+. The Landrace breed had a lower infection rate than other breeds suggesting the higher resistance of this breed compared to the others (Duroc, Large White and Pietrain)

## Conclusions

In this work, we revealed useful information concerning PRRSV epidemiology and for the first time we assessed the association between PRRSV genetic variability and some pig phenotypes. Additional research targeting other PRRSV genes and using temporal, spatial and evolutionary parameters are necessary to confirm these findings. Association studies using a larger number of animals would confirm the reported results.

## Abbreviations

PRRSV: Porcine reproductive and respiratory syndrome virus; GP5: Glycoprotein 5; ADG: Average daily gain; ORF: Open reading frame

## Competing interests

The authors declare that they have no competing interests.

## Authors’ contributions

BB performed the association studies, the genetic variability analyses and drafted the manuscript. RG participated in experimental design, sequenced part of the ORF5 samples, helped in sample collection and drafted the manuscript. SC performed spatio-temporal analyses and analysis of transmission of PRRSV by semen and helped in drafting the manuscript. MC helped in sample processing and collection. ML carried out the diagnostic PCR to assess the presence/absence of the PRRSV virus. AS was responsible for the data storage and database maintenance and helped in the manuscript correction. SB conceived the study design, coordinated the overall project, helped with the analyses of the data and the drafting of the manuscript. All authors read and approved the final manuscript.

## Supplementary Material

Additional file 1 **Entropy for PRRSV ORF5 and ORF7. 1A**. Entropy for ORF5. **1B**. Entropy for ORF7. The graph was generated using BioEdit software. After aligning the PRRSV ORF5 and ORF7 sequences, we calculated the entropy at each position of the two alignments (ORF5 and ORF7). X axis: alignment position. Y axis: entropy values. Entropy gives a measure of uncertainty at each position relative to other positions. If the nucleotidic base is the same in all sequences at a specific position the entropy takes the value 0. Entropy reach its maximum variability when at a specific position there are four possibilities for each position (A, G, C or T) and each occurs at with a frequency of 0.25. Click here for file
