# Supplementary figures and images for: Impact of genetic variation and geographic distribution of porcine reproductive and respiratory syndrome virus on infectivity and pig growth
Source: BMC Vet Res. 2013 Mar 27;9:58. doi: 10.1186/1746-6148-9-58 (PMC3762063; doi:10.1186/1746-6148-9-58)

## Slide 1
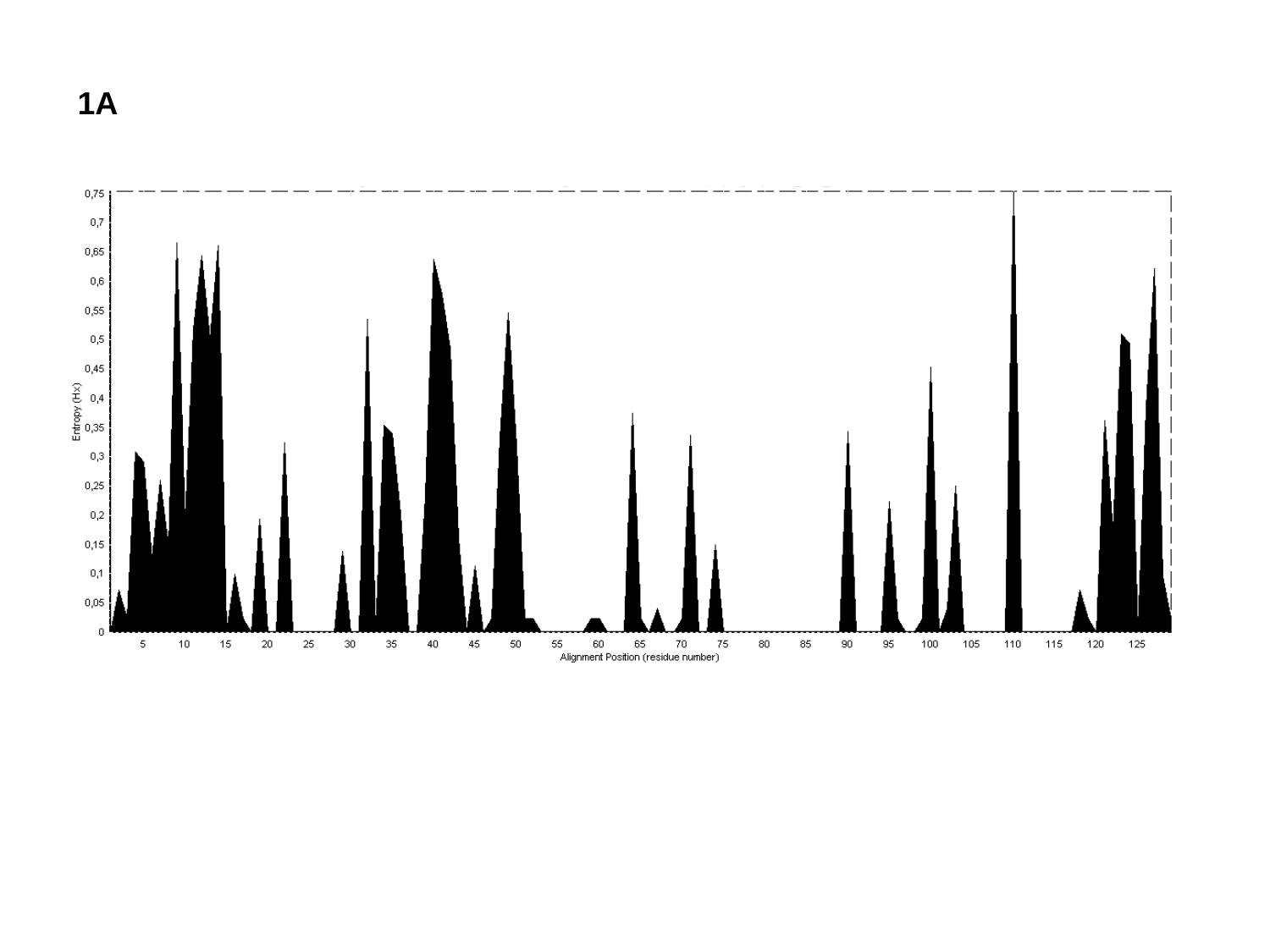

1A

## Slide 2
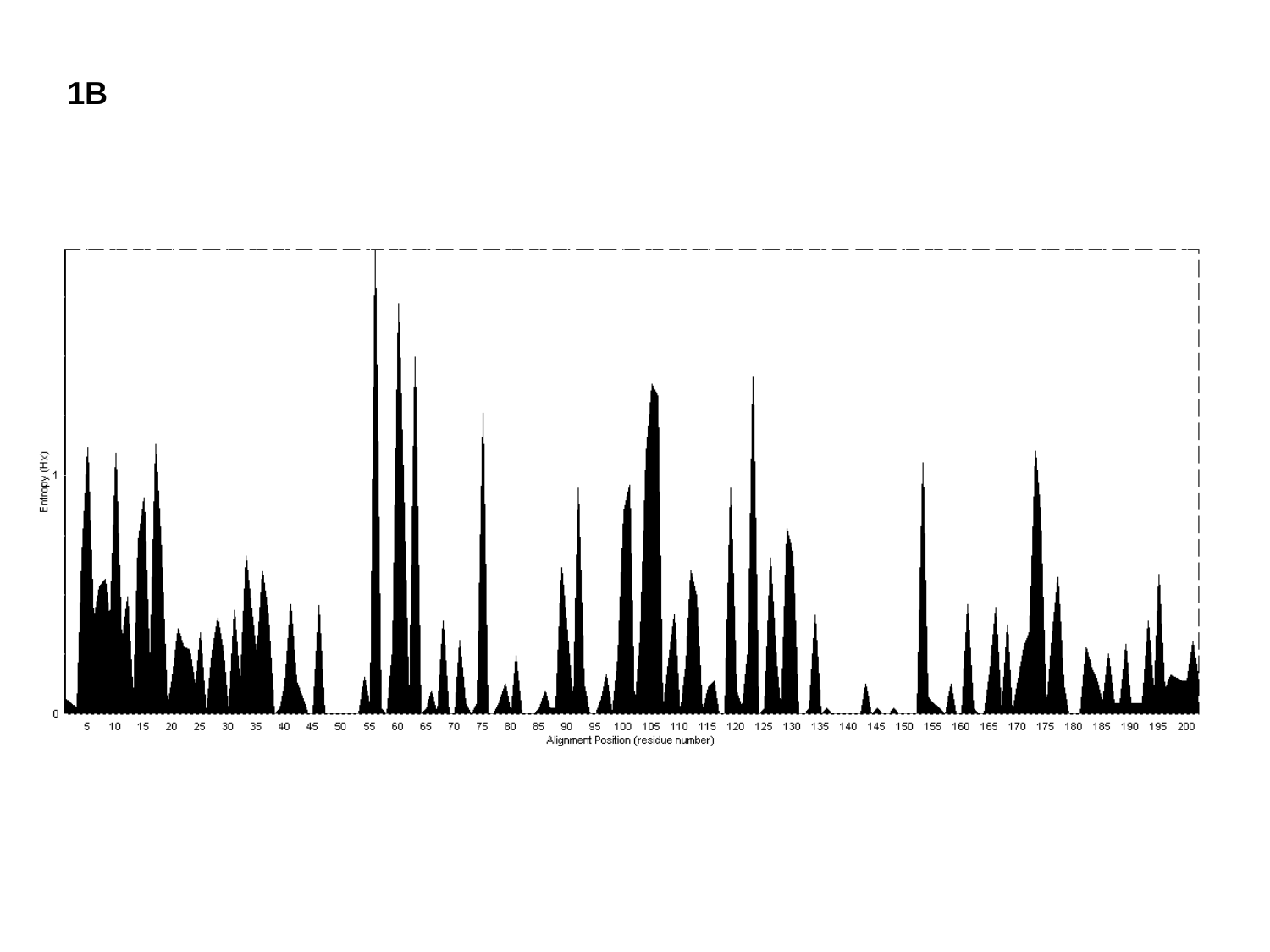

1B

Supplement: Additional file 1 — Entropy for PRRSV ORF5 and ORF7. 1A. Entropy for ORF5. 1B. Entropy for ORF7. The graph was generated using BioEdit software. After aligning the PRRSV ORF5 and ORF7 sequences, we calculated the entropy at each position of the two alignments (ORF5 and ORF7). X axis: alignment position. Y axis: entropy values. Entropy gives a measure of uncertainty at each position relative to other positions. If the nucleotidic base is the same in all sequences at a specific position the entropy takes the value 0. Entropy reach its maximum variability when at a specific position there are four possibilities for each position (A, G, C or T) and each occurs at with a frequency of 0.25. [file 1746-6148-9-58-S1.ppt]
